# Cardiovascular and cerebrovascular responses to urodynamics testing after spinal cord injury: The influence of autonomic injury

**DOI:** 10.3389/fphys.2022.977772

**Published:** 2022-09-16

**Authors:** Inderjeet S. Sahota, Vera-Ellen M. Lucci, Maureen S. McGrath, H. J. C. (Rianne) Ravensbergen, Victoria E. Claydon

**Affiliations:** ^1^ Department of Biomedical Physiology and Kinesiology, Simon Fraser University, Burnaby, BC, Canada; ^2^ International Collaboration on Repair Discoveries (ICORD), University of British Columbia, Vancouver, BC, Canada

**Keywords:** spinal cord injury, autonomic dysreflexia, urodynamics, cerebral blood flow, cardiac arrhythmia

## Abstract

Autonomic dysfunction is a prominent concern following spinal cord injury (SCI). In particular, autonomic dysreflexia (AD; paroxysmal hypertension and concurrent bradycardia in response to sensory stimuli below the level of injury) is common in autonomically-complete injuries at or above T6. AD is currently defined as a >20 mmHg increase in systolic arterial pressure (SAP) from baseline, without heart rate (HR) criteria. Urodynamics testing (UDS) is performed routinely after SCI to monitor urological sequelae, often provoking AD. We, therefore, aimed to assess the cardiovascular and cerebrovascular responses to UDS and their association with autonomic injury in individuals with chronic (>1 year) SCI. Following blood draw (plasma norepinephrine [NE]), continuous SAP, HR, and middle cerebral artery blood flow velocity (MCAv) were recorded at baseline (10-minute supine), during standard clinical UDS, and recovery (10-minute supine) (*n* = 22, age 41.1 ± 2 years, 15 male). Low frequency variability in systolic arterial pressure (LF SAP; a marker of sympathetic modulation of blood pressure) and cerebral resistance were determined. High-level injury (≥T6) with blunted/absent LF SAP (<1.0 mmHg^2^) and/or low plasma NE (<0.56 nmol•L^−1^) indicated autonomically-complete injury. Known electrocardiographic markers of atrial (p-wave duration variability) and ventricular arrhythmia (T-peak–T-end variability) were evaluated at baseline and during UDS. Nine participants were determined as autonomically-complete, yet 20 participants had increased SAP >20 mmHg during UDS. Qualitative autonomic assessment did not discriminate autonomic injury. Maximum SAP was higher in autonomically-complete injuries (207.1 ± 2.3 mmHg) than autonomically-incomplete injuries (165.9 ± 5.3 mmHg) during UDS (*p* < 0.001). HR during UDS was reduced compared to baseline (*p* = 0.056) and recovery (*p* = 0.048) only in autonomically-complete lesions. MCAv was not different between groups or phases (all *p* > 0.05). Cerebrovascular resistance index was increased during UDS in autonomically-complete injuries compared to baseline (*p* < 0.001) and recovery (*p* < 0.001) reflecting intact cerebral autoregulation. Risk for both atrial and ventricular arrhythmia increased during UDS compared to baseline (*p* < 0.05), particularly in autonomically-complete injuries (*p* < 0.05). UDS is recommended yearly in chronic SCI but is associated with profound AD and an increased risk of arrhythmia, highlighting the need for continued monitoring during UDS. Our data also highlight the need for HR criteria in the definition of AD and the need for quantitative consideration of autonomic function after SCI.

## 1 Introduction

Autonomic dysfunction is widespread following spinal cord injury (SCI) and profoundly impacts morbidity and mortality (Garshick et al., 2005). Injury to descending spinal autonomic pathways impairs sympathetic control of the heart and the vasculature, with the degree of impairment dependent on both the level and severity of spinal injury (Claydon and Krassioukov, 2007; Lucci et al., 2021a). Of particular concern, individuals with high-level SCI (injuries at or above the sixth thoracic spinal level [T6]) are at risk for a unique form of cardiovascular dysfunction known as autonomic dysreflexia (AD; paroxysmal hypertension in response to sensory stimuli, particularly visceral stimuli, below the level of spinal injury that persists until removal of the stimulus) (Blackmer, 2003; Krassioukov and Claydon, 2006; Llewellyn-Smith, Weaver and Keast, 2006). AD can be life-threatening and has been associated with conditions linked to impaired cerebrovascular haemodynamic control such as headache, blurred vision, and stroke (Pan et al., 2005; Collins, Rodenbaugh and DiCarlo, 2006; Elliott and Krassioukov, 2006), as well as with increased risk for cardiac arrhythmia (Ravensbergen et al., 2012).

In addition to these periods of episodic hypertension, autonomic dysfunction after SCI is also associated with resting hypotension (Chao and Cheing, 2008; Krassioukov et al., 2009), with profound orthostatic hypotension (OH, marked reductions in blood pressure when assuming an upright position) (Sahota et al., 2012), that is linked to decreased cerebral perfusion and subsequent cognitive decline (Jegede et al., 2010). The impact of these repeated swings in blood pressure, from profound hypotension to hypertension, multiple times in a day, on the cerebral circulation is only recently being realised (van der Scheer et al., 2017; Phillips et al., 2018; Currie et al., 2019). Previously, we identified impaired cerebrovascular responses to orthostatic stress in people with high level SCI (Sahota et al., 2012) that were associated with symptoms of OH. However, whether injury to spinal autonomic pathways impairs cerebral autoregulation is unclear; dynamic cerebral autoregulation is impaired during hypotensive stimuli (Sahota et al., 2012) but it is not clear whether any effects of SCI on cerebral autoregulatory responses vary in response to hypertension compared to hypotension (Phillips et al., 2013; van der Scheer et al., 2018).

Urodynamic studies (UDS) are used to assess bladder function after SCI, because neurogenic bladder dysfunction is common due to disruption to the neural coordination required for micturition (Karlsson, 2006; Wrathall and Emch, 2006; Stoffel, 2016). Like most visceral stimuli, UDS is a potent trigger for AD in those with high-level SCI (Curt et al., 1997; Huang et al., 2011; Koyuncu and Ersoz, 2017) and yet cardiovascular monitoring is not typically incorporated during UDS. UDS also represents a controlled stimulus that permits the assessment of cardiovascular autonomic control and cerebrovascular regulation during AD.

SCI is a heterogeneous condition and, despite the risk of AD in high-level SCI, the presence and magnitude of response cannot be anticipated based on the standard clinical quantification of SCI using the American Spinal Injury Association (ASIA) Impairment Scale (AIS), which focuses on motor and sensory deficits and does not include a quantitative assessment of autonomic function (Alexander et al., 2009). Previous work to quantify the progression of autonomic dysfunction following SCI and its effects on cardiovascular control has provided strong rationale for the evaluation of injury to descending spinal autonomic pathways using simple quantitative measures of autonomic function because they are more strongly related to measures of cardiovascular and cerebrovascular function (Claydon and Krassioukov, 2007; Ravensbergen et al., 2012; Sahota et al., 2012; Lucci et al., 2020; Lucci et al., 2021a). More recently, an addition to the AIS, the International Standards to document Autonomic Function following SCI (ISAFSCI) has been developed, but it is not yet known whether this addition correlates well with the severity of cardiovascular or cerebrovascular dysfunction after SCI (Wecht et al., 2021).

Therefore, this study aimed to assess 1) the impact of UDS on cardiovascular control and the severity of any associated AD; 2) the associations between markers of autonomic injury and cardiovascular and cerebrovascular responses during UDS in individuals with chronic spinal cord injury.

## 2 Methods

### 2.1 Ethics approval

This study was approved by the Department Of Research Ethics at Simon Fraser University and conforms to the principles outlined in the Declaration of Helsinki (World Medical Association, 2008). All participants provided written informed consent, followed by a brief medical history and screening for possible urinary tract infection to confirm eligibility for the study.

### 2.2 Participants

Eligible participants were healthy individuals >18 years of age living with a chronic (>1 year) traumatic spinal cord injury. Individuals were excluded from the study if they had cardiovascular disease, neurological disease (apart from SCI), diabetes mellitus, active pressure sores, urinary tract infections and/or were ventilator dependent. For statistical comparisons, participants were grouped as those with profound autonomic injury (autonomically-complete) and those without (autonomically-incomplete), as outlined below. Level and severity of injury to motor and sensory pathways was determined from the AIS scale (Rupp et al., 2021).

### 2.3 Urodynamics assessment

In this cross-sectional study, cardiovascular and cerebrovascular responses during UDS were assessed in the morning in a temperature-controlled laboratory. Participants were instrumented with a standard 3-lead electrocardiogram (ECG; lead II) to assess heart rate and rhythm, and continuous beat-to-beat blood pressure monitoring (Finometer Pro, Finapres Medical Systems, Amsterdam, Netherlands) of systolic (SAP) and diastolic (DAP) arterial pressure at a sampling frequency of 1 KHz. Partial pressures of end-tidal carbon dioxide (P_ET_CO_2_) and oxygen (P_ET_O_2_) were determined on a breath-by- breath basis (O_2_Cap; Oxigraf, Inc., Mountain View, CA) using a nasal cannula. Mean (MCAv_m_), systolic (MCAv_s_), and diastolic (MCAv_d_) middle cerebral artery (MCA) blood flow velocities were continuously recorded bilaterally using transcranial Doppler ultrasound (TCD; DWL Doppler-Box; Compumedics, Hamburg, Germany) with a 2 MHz probe that was held in place with a headband to ensure a constant angle of insonation. MCAv were computed by integrating the waveforms over each heartbeat. As there were no differences between sides from the bilateral MCA recordings (paired t test), cerebral blood flow data were averaged for the two vessels.

**FIGURE 1:**
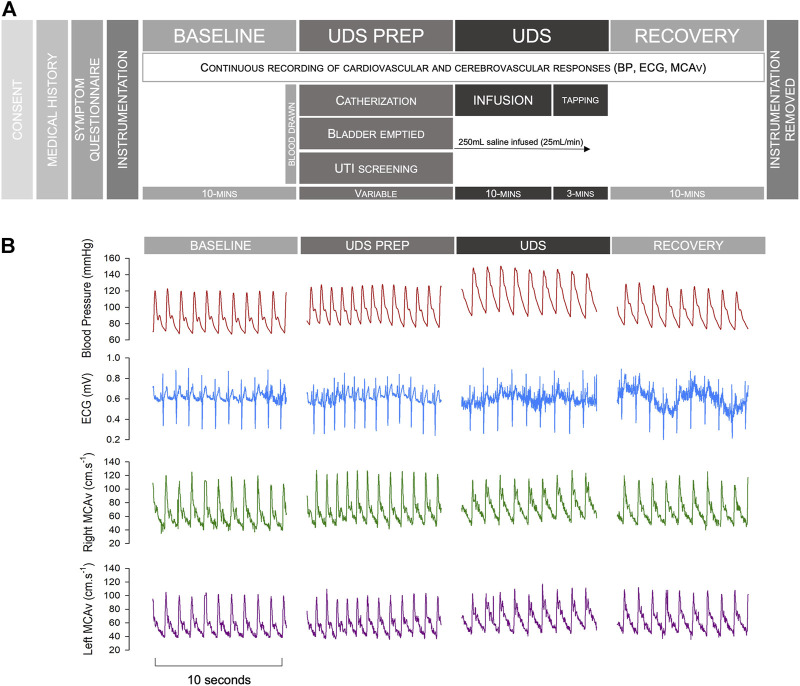
Study protocol and example traces. **(A)** Study protocol. **(B)** Representative example traces from an individual with an autonomically-complete SCI during different phases of testing. Note that occasional noise on the ECG tracing reflects muscle spasm in this individual with severe spasticity, particularly during AD. Abbreviations: BP, blood pressure; ECG, electrocardiogram; MCAv, middle cerebral artery blood flow velocity; UDS, urodynamics study; UTI, urinary tract infection.

After a 10-minute baseline period of supine rest, participants underwent a standard clinical UDS. Participants were catheterized, and their bladder was emptied. Pressure transducers were placed in the bladder and rectum. After emptying, 250 ml of warm sterile water was infused into the bladder at a rate of 25 ml/min for 10 min. If this stimulus was sufficient to trigger AD, the test was terminated. However, if this stimulus did not provoke AD, bladder percussions were performed for 3 min. The bladder was then emptied, the catheter was removed, and data were collected for a further 10-minute supine recovery phase. Detrusor pressures were calculated by subtracting abdominal pressure (estimated as rectal pressure) from intravesical pressure. AD was defined according to the current Paralysed Veterans Associated (PVA) Clinical Practice Guidelines (Consortium for Spinal Cord Medicine, 2002) and ISAFSCI criteria (Wecht et al., 2021) as a rise in SAP >20 mmHg from baseline during UDS. Data were stored for offline analysis. A schematic outlining the experimental protocol and showing representative example traces from one participant can be found in Figure 1. Blood pressure can be extremely labile during episodes of AD, with sudden and profound increases in blood pressure. For safety reasons, testing was stopped early, and the recovery phase initiated at the participant’s request (e.g., if symptomatic AD occurred) (*n* = 4), or if a blood pressure spike exceeding 200 mmHg was observed (even if asymptomatic) (*n* = 1).

Both SAP and DAP were extracted from arterial waveforms using LabChart (Lab Chart 8, ADInstruments, Colorado Springs, CO). Heart rate (HR) was determined from the interval between successive ECG R-R peaks (RRI) (HR [bpm] 60/RRI [s]). Mean arterial pressure (MAP) (mmHg) was calculated as DAP +1/3 (SAP-DAP). Cerebral MAP (CMAP) was calculated as MAP (mmHg)—(height difference between the temple and the apex of the heart [cm]/1.36). Stroke volume (SV) and cardiac output (CO) were determined using Modelflow (Wesseling et al., 1993), and total peripheral resistance (TPR) was calculated as MAP/CO. The cerebrovascular resistance index was defined as CMAP divided by MCAv_m_.

All values during baseline and recovery phases were averaged over 60s. During UDS (including catheterization, infusion, and removal of equipment) data were averaged over 10-beat intervals in order to faithfully capture the labile cardiovascular responses associated with bouts of AD. Responses during the maximal AD response (the peak blood pressure recorded during the UDS stimulus) were also quantified. The overall burden of AD was also determined by expressing the associated changes in SAP cumulatively over the AD exposure time and then quantifying the area under the curve (AUC). For each participant we expressed SAP as an absolute change from baseline values, and subsequently integrated the area of the SAP curve over the first 300 heart beats (to ensure a standardised data length for all participants) during the UDS period. The cumulative sum of the integrated area was than calculated for each person. Beat-to-beat data were used to determine cerebral autoregulation, baroreflex sensitivity, and blood pressure and MCAv_m_ variability.

### 2.4 Assessment of autonomic injury

Severity of autonomic dysfunction was assessed quantitatively using resting supine plasma norepinephrine (NE) concentrations and low frequency oscillations in blood pressure variability (LF SAP) (Claydon and Krassioukov, 2007). We also evaluated qualitative measures of cardiovascular dysfunction based on the clinical evaluation proposed in the ISAFSCI standards (Wecht et al., 2021).

#### 2.4.1 Plasma norepinephrine

An intravenous catheter was inserted into an antecubital vein prior to testing. A 10 ml venous blood sample was taken after baseline, while supine. These samples were centrifuged for 10 min at −3°C and 3,000 rpm, after which the plasma fraction was stored in a freezer at −80°C for subsequent analysis. Analyses were performed at the Vancouver General Hospital, Department of Pathology and Laboratory Medicine. To assess autonomic function, absolute NE was used, as described previously (Claydon and Krassioukov, 2007; Sahota et al., 2012).

#### 2.4.2 Blood pressure variability and baroreflex sensitivity

Autoregressive monovariate models were fitted to time series generated from 10-min of beat-to-beat SAP waveforms at baseline to determine blood pressure variability, as per our standard approach (Gulli G. E. et al., 2003; Claydon and Krassioukov, 2007). Occasional ectopic beats were removed using linear interpolation of adjacent normal beats, and significant trends were removed by subtracting the best polynomial function fitted to the data using low pass filtering (Gulli G. E. et al., 2003). We quantified the low frequency (LF) oscillations in SAP (which occur between 0.04 and 0.15 Hz) because they have been shown to directly reflect sympathetic control of the vasculature and to provide a robust indicator of autonomic completeness of injury to spinal autonomic pathways in both humans and in animal models of SCI (Inskip et al., 2009; Lucci et al., 2021a). The central frequency, as well as both absolute and percent power of the LF band were determined by computation of the residuals (Johnsen and Andersen, 1978). Additionally, through the use of autoregressive cross-spectral (bivariate) analyses, we determined baroreflex sensitivity as the transfer function gain [ms•mmHg^−1^] between SAP and RRI time series in the LF range, to assess the impact of SCI and UDS on cardiac baroreflex control (Gulli G. et al., 2003).

SCI was determined to be autonomically-complete if injury was at or above T6 and the individual had either NE < 0.56 nmol•L^−1^ and/or an LF SAP <1.0 mmHg^2^ at baseline. We have previously shown these values to have the highest sensitivity and specificity to discriminate autonomic injury after SCI (Claydon and Krassioukov, 2007).

#### 2.4.3 ISAFSCI evaluation

ISAFSCI criteria for cardiovascular autonomic dysfunction were evaluated as follows: 1) bradycardia (HR ≤ 60 bpm); 2) tachycardia (HR ≥ 100 bpm); 3) systolic hypotension (SAP≤90 mmHg); 4) diastolic hypotension (DAP≤60 mmHg); 5) systolic OH (decrease in SAP within 15 min of transition from a supine to a seated position of ≥20 mmHg); 6) diastolic OH (decrease in DAP within 15 min of transition from a supine to a seated position of ≥10 mmHg); 7) autonomic dysreflexia (increase in SAP ≥20 mHg during UDS). Participants were classified as being ISAFSCI+ if they met one or more of these criteria (Vaccaro et al., 2022) and their ISFASCI score was calculated as the sum of the number of criteria met (Vaccaro et al., 2022). Note that we did not have data on orthostatic hypotension for two participants, and therefore could not determine ISAFSCI status for these two individuals, one with an autonomically-complete lesion, and one with an autonomically-incomplete lesion.

### 2.5 ECG markers of arrythmia risk

Standard ECG markers for risk of cardiac arrhythmia (variabilities of p-wave duration [PWD], QT interval corrected for HR using Bazett’s method [QTc] (Bazett, 1920), and Tpeak-Tend interval [T_peak_-T_end_] as well as QT variability index [QTVI]) were determined at baseline and maximum pressures during UDS from 100 heart beats (to ensure optimal analytical quality) using customised software (LabView 2010; National Instruments). Using this customised software, the R-peaks are determined from the ECG by taking the maximum amplitude of the derivate of the ECG signal from the point of zero slope. The T-peak is then determined from the maximum value of the third order polynomial applied to a selection of data preceding the R-peak, while the T-end is determined from the point of intersection for two regression lines, one starting at T-peak, and the other starting from a specified location after T-peak. Q was determined from the minimum value of the third order polynomial applied to a selected section of data preceding the R-peak. The P-peak was determined from the maximum point of a polynomial regressionof the 20 ms of data preceding the Q-wave. The P-end was identified from the point at which regressions applied to the section between the P-peak and Q-waves intersect. (Ravensbergen et al., 2012; Lucci et al., 2021a; Lucci et al., 2021b). The total variabilities for each ECG interval parameter were determined as the standard deviation squared while QTVI was determined using the following formula: $QTVI=log\;10\;\frac{\left(QT_{v}/QT_{m}^{2}\right)}{\left(RR_{v}/RR_{m}^{2}\right)}$ , where v and m denote the variability and the mean, respectively (La Fountaine et al., 2011; Porta et al., 2011). QTVI is typically a negative number, with a more positive (closer to zero) number indicating a greater risk of ventricular arrythmia.

### 2.6 Symptoms of autonomic dysfunction questionnaire

Severity of symptoms of AD and OH during activities of daily living were determined by questionnaire, as previously described (Sahota et al., 2012; Lucci et al., 2021a). Higher reported values indicate increased symptom frequency and severity.

### 2.7 Cerebral autoregulation

Both static and dynamic cerebral autoregulatory control were assessed at baseline and during UDS.

#### 2.7.1 Static cerebral autoregulation

Static cerebral autoregulate (sCA) was determined at baseline and during UDS from the correlation coefficient and gradient (slope) describing the relationship between MCAv_m_ and CMAP (MCAv_m_/CMAP) at various steady-state levels of CMAP taken from 10 s averages at one-minute intervals throughout testing. An increased gradient of this relationship indicates impaired autoregulation whereby small changes in pressure provoke marked changes in flow. Similarly, an increased correlation implies a strong relationship between CMAP and MCAv_m_ and thus impaired autoregulation.

#### 2.7.2 Dynamic cerebral autoregulation

Dynamic cerebral autoregulation (dCA) was determined at baseline and during UDS using autoregressive cross-spectral analyses of time series generated from continuous CMAP and MCAv_m_ recordings in the low-frequency (0.04–0.15 Hz) range (Claassen et al., 2016). Values from the VLF range were excluded because the relationship between CMAP and MCAv_m_ is not linear at these frequencies. We also excluded values in the HF range because the physiological relevance in the context of cerebral autoregulation at this frequency has been questioned (Panerai, Robinson and Minhas, 2019; Reehal et al., 2021), and the altered respiratory behaviour after SCI might impact interpretation of HF responses (Lucci et al., 2021a; Reehal et al., 2021). From these analyses, transfer function gain (TFG [cm.sec^−1^•mmHg^−1^]; sensitivity or magnitude of response), phase (deg; time delay), and coherence (strength of the relationship between variables, where 0 indicates no relationship and 1 indicates complete interdependence) were determined. As with blood pressure variability analyses, all signals were visually analysed, with artefacts and significant trends removed as described above. Increases in TFG indicated impaired autoregulation, whereby changes in CMAP elicit larger changes in MCAv_m_. Decreased phase suggests reduced autoregulatory function, as changes in CMAP rapidly induce changes in MCAv (Panerai et al., 2018).

### 2.8 Statistical analyses

Statistical analyses were performed using Sigmaplot 14 (Systat Software Inc., San Jose, CA). Data were tested for normality and parametric or non-parametric assumptions were used as appropriate. Differences in participant characteristics were compared using unpaired t-tests. Comparisons of cardiovascular and cerebrovascular outcomes and symptoms between groups (autonomically-complete and autonomically-incomplete) and test phases (baseline, during UDS, and recovery) were performed using one-way ANOVA (where either autonomic completeness or test phase was not considered) or two-way repeated measures ANOVA (when both autonomic completeness and test phase were considered together), where appropriate, and tested for normality using the Shapiro-Wilk test. Where datasets were incomplete due to loss of data resulting from signal noise, two-way ANOVAs were used in the absence of repeated measures. Spearman rank (for nonparametric data) and Pearson (for parametric data) correlations, as well as linear regressions were used to assess the relationships between variables. AD burden based on cumulative AUC was evaluated using the two-AUC analysis function in GraphPad Prism (Prism 8, San Diego, CA). Statistical significance was assumed at *p* < 0.05. Data are represented as mean ± standard error (SEM), unless otherwise stated.

## 3 Results

### 3.1 Demographic and injury information

We tested 22 individuals (15 male) who had injuries ranging from C4 to L3, and a range of motor and sensory injuries (A to D) based on AIS scores. Participants were 41.1 ± 2.3 years of age at time of testing and had an average duration of injury of 14.0 ± 2.4 years. While 16 participants were at risk for developing AD (injuries ≥ T6), using our criteria for autonomic injury, 9 participants were determined to have autonomically-complete lesions based on autonomic function testing. As expected, individuals with autonomically-complete lesions had lower LF SAP, and lower plasma NE. They also had higher AD symptom scores. However, the ISAFSCI criteria failed to discriminate groups with different severities of autonomic completeness of injury, with no difference in ISAFSCI scores between subgroups, and all participants with autonomically-complete lesions, and 92% of those with autonomically-incomplete lesions meeting ISAFSCI+ criteria, including those with injury levels below T6. Because ISAFSCI+ status did not discriminate between participants, further analysis between ISAFSCI+ and ISAFSCI- subgroups were not possible. Information on participant demographics and injury characteristics (including autonomic injury classification) can be found in Table 1.

**TABLE 1:** Participant characteristics.

Participant characteristics	
Sample size (M/F)	22 (15/7)
Age (years)	41.1 ± 2.3
Injury characteristics	
Duration of injury (years)	14.0 ± 2.4
Autonomically-complete	9 (41%)
Level of injury	
Cervical	13 (59%)
High-thoracic (T1-T6)	3 (14%)
Low (T7 and below)	6 (27%)
AIS score	
A	12 (55%)
B	5 (23%)
C	4 (18%)
D	1 (4%)
LF SAP (mmHg^2^)	
Autonomically-complete	1.13 ± 0.42
Autonomically-incomplete	6.64 ± 2.36*
Plasma NE (nmol.L^−1^)	
Autonomically-complete	0.28 ± 0.05
Autonomically-incomplete	3.77 ± 0.98*
ISAFSCI+	
Autonomically-complete	8/8 (100%)
Autonomically-incomplete	11/12 (92%)
ISAFSCI score	
Autonomically-complete	2.5 ± 0.4
Autonomically-incomplete	1.6 ± 0.3
AD Symptom Score	
Autonomically-complete	27.57 ± 3.24
Autonomically-incomplete	15.23 ± 3.86*
AD PVA blood pressure criteria met	
Autonomically-complete	8/8 (100%)
Autonomically-incomplete	11/12 (92%)

Data are presented as mean ± SEM or n (%) as appropriate. Statistical significance: * denotes statistical difference between groups. Abbreviations: AIS, American Spinal Injury Association (ASIA) Impairment scale; ISAFSCI, International Standards to document Autonomic Function after SCI; F, female; M, male; LF SAP, low frequency systolic arterial pressure variability; NE, norepinephrine; AD, autonomic dysreflexia; PVA, Paralyzed Veterans of America.

### 3.2 Cardiovascular responses to UDS

**FIGURE 2:**
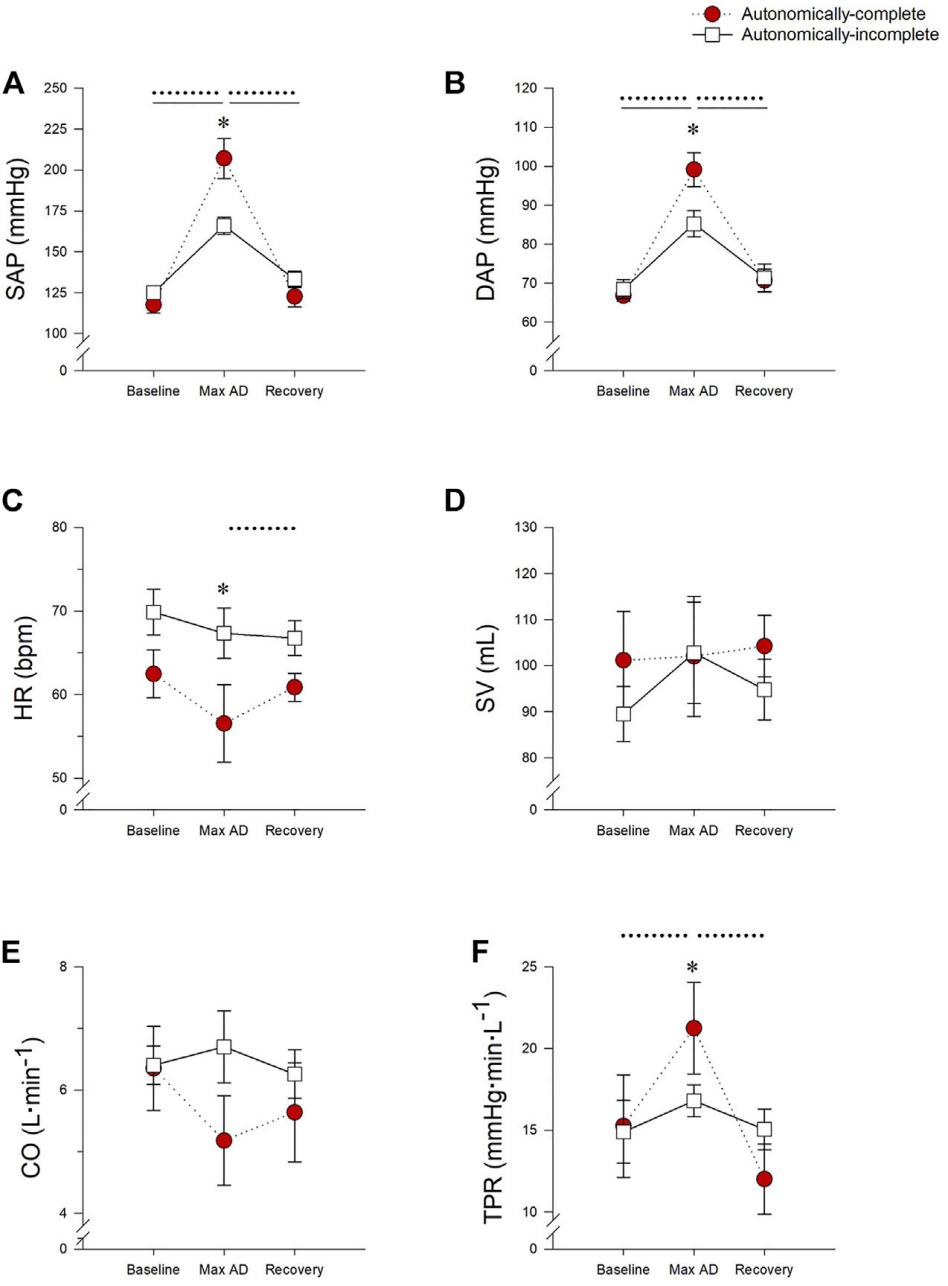
Impact of autonomic completeness of injury on cardiovascular responses at the point of the maximum blood pressure increase during urodynamics testing. **(A)** Systolic arterial pressure (SAP) was increased during urodynamics testing (UDS) compared to baseline and recovery for both those with autonomically-complete (*p* < 0.001 for both comparisons) and autonomically-incomplete (*p* < 0.001 for both comparisons) injuries. During UDS SAP was also significantly increased in those with autonomically-complete injuries compared to those with autonomically-incomplete injuries (*p* < 0.001). **(B)** Diastolic arterial pressure was also increased in those with autonomically-complete injuries compared to autonomically-incomplete injuries during UDS (*p* = 0.004), although both groups had increased DAP during UDS compared to baseline and recovery (all *p* < 0.001). **(C)** In those with autonomically-complete injuries, heart rate (HR) was significantly decreased during UDS compared to those with autonomically-incomplete injuries (*p* = 0.016). HR also tended to be decreased during UDS compared to baseline (*p* = 0.056) and was significantly reduced in recovery (*p* = 0.048) in those with autonomically-complete lesions. Stroke volume **(D)** and cardiac output **(E)** were not significantly different in the different phases of the test, or between groups (*p* > 0.05 for all). **(F)** In those with autonomically-complete injuries, total peripheral resistance (TPR) was significantly increased during UDS compared to those with autonomically-incomplete injuries (*p* = 0.013). TPR was also significantly increased during UDS compared to baseline (*p* = 0.006) and recovery (*p* < 0.001) in those with autonomically-complete lesions. Statistical significance: * denotes statistical difference between groups; dashed line (---) denotes changes over time in autonomically-complete group; solid line (–) denotes changes over time in autonomically-incomplete group. Abbreviations: AD, autonomic dysreflexia; SAP, systolic arterial pressure; DAP, diastolic arterial pressure; HR, heart rate; SV, stroke volume; CO, cardiac output; TPR, total peripheral resistance.

Cardiovascular responses to UDS, taken at the point of maximal AD, can be found in Figure 2 and Supplemental Table S1. Maximum SAP during UDS was higher in all participants compared to baseline (*p* < 0.001) and recovery phases (*p* < 0.001). However, the max SAP was significantly higher in those with autonomically-complete injuries (207.1 ± 12.3 mmHg) compared to those with autonomically-incomplete injuries (165.9 ± 5.3 mmHg) during UDS (*p* < 0.001) (Figure 2A). Similarly, max DAP increased during UDS in both groups compared to baseline (*p* < 0.001) and recovery (*p* < 0.001), with a significantly higher max DAP in those with autonomically-complete (99.2 ± 4.4 mmHg) versus autonomically-incomplete (85.3 ± 3.4 mmHg) injuries during UDS (*p* = 0.004) (Figure 2B). Not surprisingly, these increased pressures were accompanied by a reduction in HR during UDS in the autonomically-complete group compared to the autonomically-incomplete group (*p* = 0.016), presumably due to the vagally-mediated bradycardia induced during AD (Figure 2C). The HR during UDS tended to be reduced compared to baseline (*p* = 0.056) and recovery (*p* = 0.048) only in those with autonomically-complete lesions, although the reduction relative to baseline did not quite achieve statistical significance. There were no significant differences in SV (Figure 2D) and CO (Figure 2E) between or within groups throughout testing (all *p* > 0.05). However, TPR responses were increased in those with autonomically-complete injuries during UDS compared to baseline (*p* = 0.006) and recovery (*p* < 0.001) and were significantly higher during UDS compared to those with autonomically-incomplete injuries (*p* = 0.013) (Figure 2F). There were no differences in baroreflex sensitivity between subgroups at baseline (autonomically-complete 13.3 ± 3.4 ms.mmHg^−1^, autonomically-incomplete 9.9 ± 2.4 ms.mmHg^−1^; *p* = 0.363) or during UDS (autonomically-complete 9.0 ± 2.2 ms.mmHg^−1^, autonomically-incomplete 10.0 ± 2.3 ms.mmHg^−1^; *p* = 0.785), or within those with autonomically-complete (*p* = 0.149) or autonomically-incomplete (*p* = 0.946) SCI between baseline and UDS.

### 3.3 The burden of AD

Based on the PVA criteria for the presence of AD of an increase in SAP of 20 mmHg, as one might expect, AD was induced by the UDS procedure in all participants with autonomically-complete lesions (SAP increase +89.5 ± 13.0 mmHg). Surprisingly, 11 participants with autonomically-incomplete lesions also met this criterion for induction of AD, despite having a low probability of experiencing AD based on their lesion level and severity. Of note, the increase in SAP was larger in those with autonomically-complete lesions compared to those with autonomically-incomplete injuries (+40.1 ± 5.0 mmHg, *p* < 0.001) and this reflects the higher max SAP noted above. Similarly, individuals with higher ISAFSCI scores had larger increases in SAP during UDS (r = 0.540, *p* = 0.01) although ISAFSCI was not correlated with any other metrics of AD severity or autonomic function (all *p* > 0.05).

**FIGURE 3:**
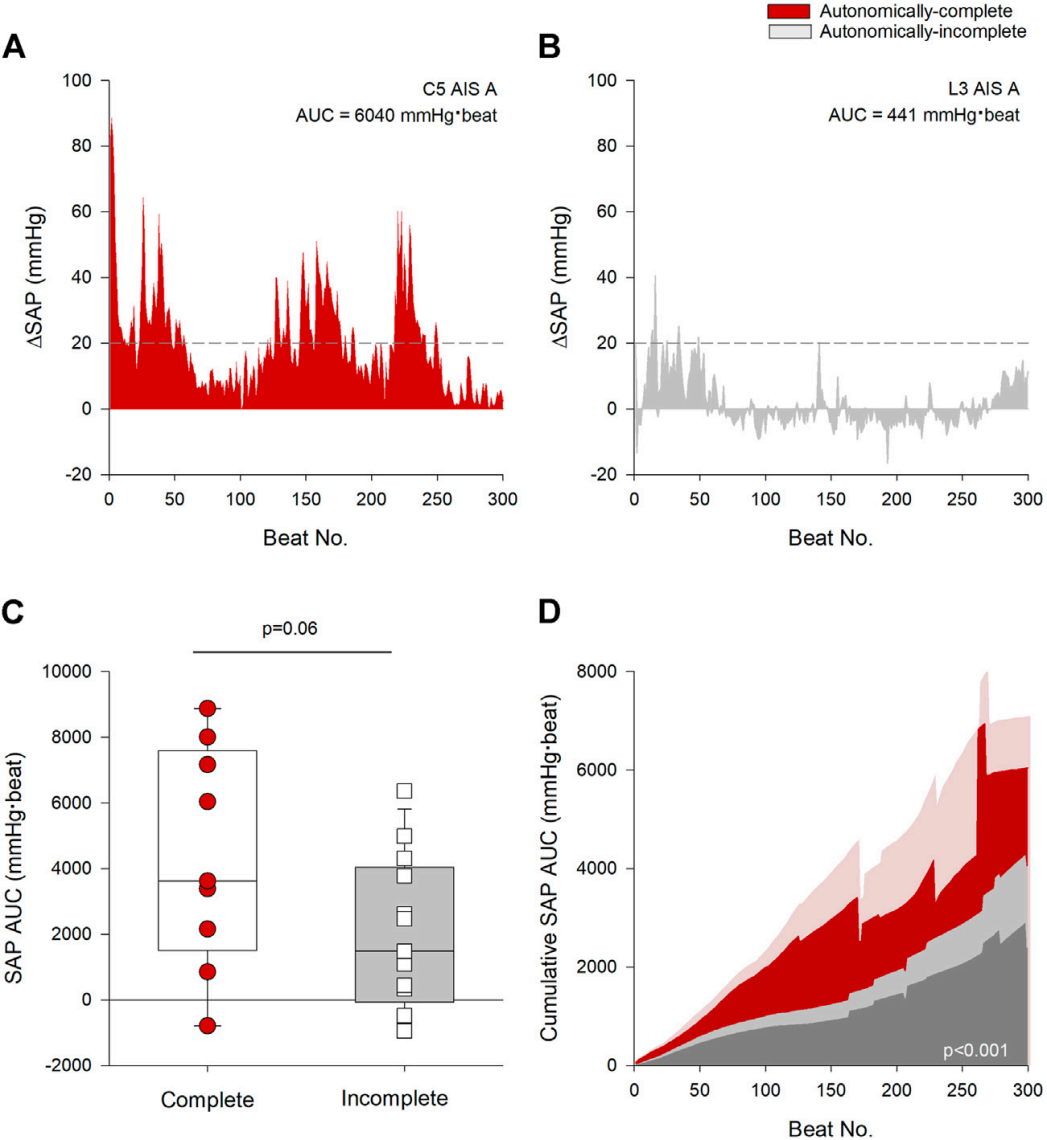
Autonomic injury was associated with greater severity and burden of AD. **(A)** Change in beat-to-beat systolic arterial pressure (SAP) recorded from an individual with a C5 AIS A autonomically-complete injury during the first 300 beats of urodynamics testing (UDS). **(B)** Change in beat-to-beat SAP recorded from an individual with an L3 AIS A autonomically-incomplete injury during the first 300 beats of UDS. Threshold criteria for the diagnosis of AD is outlined in **(A,B)** using a dashed line at +20 mmHg. **(C)** Individuals with autonomically-complete injuries tended to have increased SAP AUC compared to those with autonomically-incomplete injuries (*p* = 0.06). **(D)** Mean cumulative area under the curve (AUC) for systolic pressure over the first 300 beats during UDS by completeness of injury. Red area denotes autonomically-complete injuries and grey area denotes autonomically-incomplete injuries. Respective pale shaded areas denote corresponding standard error. Note that the AUC is significantly larger in those with autonomically-complete injuries compared to those with autonomically-incomplete injuries (*p* < 0.001). Statistical significance: straight line ( – ) denotes statistical difference between groups. Abbreviations: AD, autonomic dysreflexia; SAP, systolic arterial pressure; AUC, area under the curve; UDS, urodynamics testing.

We quantified the overall burden of AD from the AUC of the SAP response over time as shown in the representative tracings in Figures 3A,B. Note the difference in SAP AUC between the individual with the autonomically-complete injury (Figure 3A) compared to the individual with the autonomically-incomplete injury (Figure 3B). Interestingly, despite the presence of a lumbar lesion, that is, not compatible with susceptibility to AD, the individual with the autonomically-incomplete lesion does meet criteria for AD based on the PVA threshold definition (as defined by the dashed line). Overall, participants with autonomically-complete injuries tended to have an increased burden of AD during UDS compared to those with autonomically-incomplete injuries although this did not quite meet criteria for statistical significance (*p* = 0.06) (Figure 3C). Cumulative SAP AUC was greater in individuals with autonomically-complete injuries compared to those with autonomically-incomplete injuries (*p* < 0.001) (Figure 3D). Interestingly, there was a trend for the cumulative SAP AUC to be correlated with the maximal detrusor pressure during UDS (r = 0.437, *p* = 0.06) suggesting that this response may be dose-dependent.

### 3.4 Cerebrovascular responses to UDS

**FIGURE 4:**
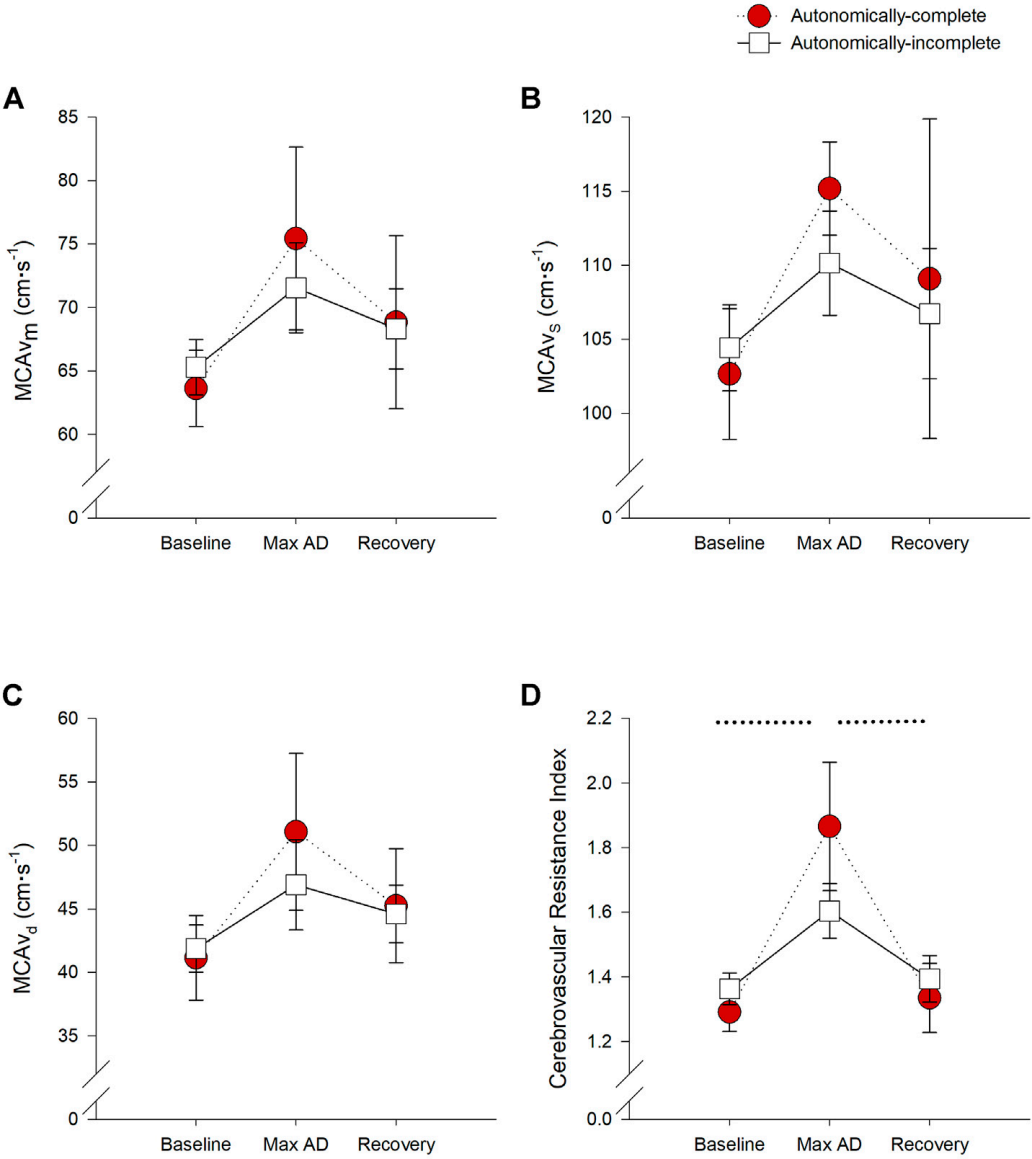
Impact of autonomic severity of injury on cerebrovascular responses during urodynamics testing. Neither mean **(A)**, systolic **(B)**, or diastolic **(C)** cerebral blood flow velocities were significantly different within or between groups. However, when assessing cerebrovascular resistance **(D)**, resistance was significantly increased during UDS compared to baseline (*p* = 0.001) and recovery (*p* = 0.003) in those with autonomically-complete injuries. Additionally, cerebrovascular resistance tended to increase in those with autonomically-complete injuries compared to those with autonomically-incomplete injuries (*p* = 0.065) during UDS. Statistical significance: * denotes statistical difference between groups; dashed line (---) denotes changes over time in autonomically-complete group. Abbreviations: AD, autonomic dysreflexia; MCAv_m_, mean middle cerebral artery blood flow velocity, MCAv_s_, systolic middle cerebral artery blood flow velocity; MCAv_d_, diastolic middle cerebral artery blood flow velocity.

All measures of MCAv were not significantly different between groups or phases (all *p* > 0.05) (Figures 4A–C). However, the cerebrovascular resistance index was increased during UDS in those with autonomically-complete injuries compared to baseline (*p* < 0.001) and recovery conditions (*p* < 0.001) (Figure 4D). Of note, there were no significant differences in end tidal carbon dioxide levels between groups or test phases at any time, so we do not believe that hypocapnic vasoconstriction influenced the cerebral responses observed.

### 3.5 sCA

Measures of sCA can be found in Table 2. sCA was not significantly different between those with autonomically-complete or autonomically-incomplete lesions, and was not significantly affected by the presence of AD. There were no differences in the correlation coefficient or gradient describing the efficacy of sCA either between or within groups (all *p* > 0.05).

**TABLE 2:** Measures of static and dynamic cerebral autoregulation at baseline and during urodynamics.

	Baseline	Urodynamics	Time	Completeness	Interaction
Static Cerebral Autoregulation					
Correlation					
Autonomically-complete	0.38 ± 0.06	0.44 ± 0.12	0.084	0.203	0.507
Autonomically-incomplete	0.39 ± 0.06	0.66 ± 0.11			
Gradient (cm.s^−1^.mmHg^−1^)					
Autonomically-complete	0.81 ± 0.24	0.30 ± 0.09	0.242	0.633	0.322
Autonomically-incomplete	0.65 ± 0.20	0.60 ± 0.16			
Dynamic Cerebral Autoregulation					
MCAv_m_ Total Power (cm.s^−1^)^2^					
Autonomically-complete	38.4 ± 16.4	46.8 ± 9.4	0.187	0.291	0.659
Autonomically-incomplete	24.4 ± 5.3	41.0 ± 6.8			
CMAP Total Power (mmHg^2^)					
Autonomically-complete	4.5 ± 0.9	22.4 ± 4.7 ^B^	**0.042**	0.970	**0.004**
Autonomically-incomplete	15 ± 4.1*	11.6 ± 1.6*			
Low frequency					
Frequency (Hz)					
Autonomically-complete	0.12 ± 0.02	0.14 ± 0.01	0.693	0.376	0.063
Autonomically-incomplete	0.13 ± 0.02	0.14 ± 0.01			
Gain (cm.s^−1^.mmHg^−1^)					
Autonomically-complete	1.05 ± 0.28	0.83 ± 0.24	0.255	0.977	0.177
Autonomically-incomplete	0.91 ± 0.17	0.79 ± 0.08			
Coherence					
Autonomically-complete	0.40 ± 0.16	0.21 ± 0.09	0.118	0.777	0.223
Autonomically-incomplete	0.29 ± 0.07	0.22 ± 0.05			
Phase (deg)					
Autonomically-complete	29.9 ± 35.2	56.8 ± 16.4	0.331	0.455	0.314
Autonomically-incomplete	55.6 ± 13.0	60.0 ± 11.6			

Data are presented as mean ± SEM. Statistical significance: * represents significant difference between groups; ^B^ represents statistical difference from baseline condition. Statistical significance (*p*-values) are also presented for the main effects of time point, subgroup classification based on autonomic completeness of injury, and their interactions, where bold text indicates significant main effects.

MCAv_m_, mean middle cerebral artery blood flow velocity; CMAP, corrected mean arterial pressure.

### 3.6 dCA

Similarly, for the group as a whole, there was no effect of AD on measures of dynamic autoregulation. However, as expected given the lower blood pressure variability in those with autonomically-complete lesions, total power of CMAP (the input parameter) was reduced in those with autonomically-complete lesions at baseline (*p* = 0.047) and increased during UDS in this group only (*p* = 0.003) becoming larger than those with autonomically-incomplete lesions during UDS (*p* = 0.024). Dynamic autoregulation was unaffected by completeness of injury or the presence of AD, and there were no significant differences in measures of LF coherence, TFG, or phase between groups, or within groups in the different test phases (all *p* > 0.05). Despite low coherence, gain and phase were similar to healthy controls in the LF range (Claassen et al., 2016). Measures of dCA can be found in Table 2.

### 3.7 Symptoms of cardiovascular dysfunction

Symptoms of AD were prevalent and significantly more severe in those with autonomically-complete injuries (*p* = 0.048) (Table 1). We considered whether self-reported symptoms of AD were associated with discrete markers of autonomic function. AD symptom scores were negatively correlated with LF SAP (r = -0.527, *p* = 0.017) and tended to be negatively correlated with plasma NE, although this did not achieve our criteria for statistical significance (r = −0.531, *p* = 0.075). There was no correlation between ISAFSCI scores and AD symptom scores (r = 0.314, *p* = 0.19).

### 3.8 Effect of UDS on risk for cardiac arrythmia

ECG interval parameters and markers of arrhythmia risk were assessed at baseline and during UDS. Absolute values for all interval parameters and markers of arrhythmia risk can be found in Table 3. To allow for repeated-measures assessments, where data were missing due to noisy/unreadable ECG, they were excluded from statistical analysis.

**TABLE 3:** ECG markers for risk of cardiac arrhythmia at baseline and during urodynamics testing.

	Baseline	Urodynamics	Time	Completeness	Interaction
PWD variability (ms^2^)					
Autonomically-complete	365.9 ± 87.6	478.2 ± 65.2^B^	**<0.001**	**0.006**	0.217
Autonomically-incomplete	158.7 ± 38.6*	264.65 ± 43.3^B^*			
T_peak-_T_end_ variability (ms^2^)					
Autonomically-complete	102.4 ± 27.8	162.3 ± 101.9^B^	**0.003**	**0.029**	0.442
Autonomically-incomplete	33.8 ± 8.4	101.89 ± 23.7*			
QTc variability (ms^2^)					
Autonomically-complete	204.2 ± 50.6	259.4 ± 54.5	*0.055*	0.235	0.255
Autonomically-incomplete	169.7 ± 42.3	193.1 ± 27.6			
QTVI					
Autonomically-complete	−0.94 ± 0.16	-0.83 ± 0.15	0.505	0.864	0.719
Autonomically-incomplete	−0.92 ± 0.20	-0.88 ± 0.19			

Data are presented as mean ± SEM. Statistical significance: * represents significant difference between groups; ^B^ represents statistical difference from baseline condition. Statistical significance (*p*-values) are also presented for the main effects of time point, subgroup classification based on autonomic completeness of injury, and their interactions, where bold text indicates significant main effects and italicised text indicates main effects that did not quite meet the threshold criteria for statistical significance.

PWD, p-wave duration; T_peak-_T_end_, T peak to T end interval; QTc, QT, interval corrected for heart rate; QTVI, QT variability index.

**FIGURE 5:**
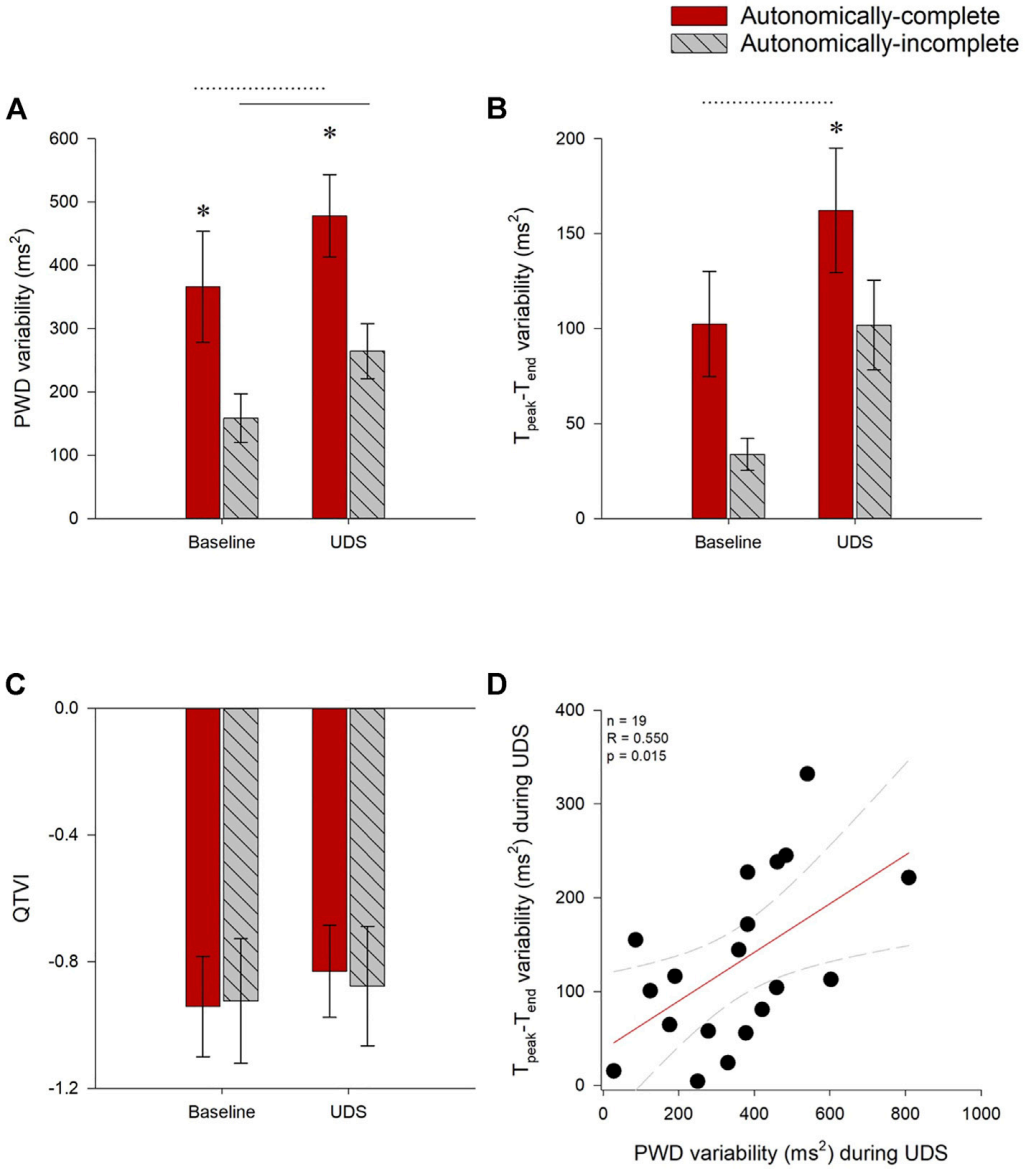
Impact of autonomic injury on electrocardiographic predictors of cardiac arrhythmia at baseline and during UDS. **(A)** P-wave duration (PWD) variability was increased in those with autonomically-complete injuries compared to those with autonomically-incomplete lesions, both at baseline (*p* = 0.017) and during UDS (*p* = 0.005). PWD variability was also increased compared to baseline in both autonomically-complete (*p* < 0.001) and autonomically-incomplete (*p* = 0.008) groups. **(B)** Tpeak-Tend interval (T_peak_-T_end_) variability tended to be increased in those with autonomically-complete lesions at baseline, but this did not quite meet criteria for statistical significance (*p* = 0.055). Tpeak-Tend interval variability was significantly increased during UDS in those with autonomically-complete (*p* = 0.021) compared to autonomically-incomplete injuries. T_peak_-T_end_ variability also increased in autonomically-complete injuries during UDS compared to baseline (*p* = 0.01). **(C)** QTVI was not different between or within groups (*p* > 0.05). **(D)** During UDS, increased PWD variability was correlated with increased T_peak_-T_end_ variability (r = 0.550, *p* = 0.015). Statistical significance: * denotes statistical difference between groups; dashed line (---) denotes changes over time in autonomically-complete group; solid line (–) denotes changes over time in autonomically-incomplete group. Abbreviations: UDS, urodynamics testing; PWD, p-wave duration; T_peak_-T_end_, Tpeak—Tend interval; QTVI, QT variability index.

For all participants, risk for atrial arrhythmia (PWD variability) increased during UDS compared to baseline (main effect *p* = 0.006), in both autonomically-complete (*p* < 0.001) and autonomically-incomplete (*p* = 0.008) injuries. However, both at baseline (*p* = 0.017) and during UDS (*p* = 0.005), PWD variability was increased in those with autonomically-complete injuries compared to those with autonomically-incomplete injuries (Figure 5A).

Ventricular arrhythmia risk (T_peak_-T_end_ variability) was increased in those with autonomically-complete injuries compared to those with autonomically-incomplete injuries (main effect *p* = 0.029), particularly during UDS (*p* = 0.021), and tended to be increased at baseline in those with autonomically-complete lesions compared to those with autonomically-incomplete lesions, although this did not quite meet criteria for statistical significance (*p* = 0.055). T_peak_-T_end_ variability was significantly increased during UDS compared to baseline (main effect *p* = 0.003) in those with autonomically-complete (*p* = 0.01) injuries (Figure 5B). However, there were no changes to the alternative metric of ventricular arrhythmia risk (QTVI) either between baseline and UDS, or between groups (Figure 5C).

We wondered whether the increased risk for atrial and ventricular arrhythmia observed in those with autonomically-complete lesions were correlated with one another. During UDS, T_peak_-T_end_ variability was positively correlated with PWD variability (r = 0.550, *p* = 0.015) (Figure 5D). T_peak_-T_end_ variability was also positively correlated with the severity of AD, inferred from the SAP AUC (r = 0.683, *p* = 0.002).

## 4 Discussion

We have confirmed previous reports that UDS, like other visceral stimuli, is a potent trigger for AD in susceptible individuals with SCI (Giannantoni et al., 1998; Huang et al., 2013; Liu et al., 2013; Lucci et al., 2020). With beat-to-beat monitoring we showed that blood pressure increases with UDS in those with autonomically-complete SCI are profound, with SAP values in excess of 200 mmHg. These blood pressure responses reflect profound increases in TPR associated with peripheral vascular sympathetic activation and consequent vasoconstriction, and are associated with concurrent bradycardia and an increased risk for both atrial and ventricular arrhythmia. In many cases these episodes of AD were highly symptomatic, although silent AD was also observed. Interestingly, cerebral blood flow did not increase significantly during these hypertensive responses, reflecting compensatory increases in the cerebrovascular resistance index with preserved static and dynamic autoregulation.

Guidelines suggest yearly UDS screening after SCI (Linsenmeyer and Linsenmeyer, 2013; Kavanagh et al., 2019), and this means that susceptible individuals living with SCI will encounter unavoidable situations during medical procedures such as UDS that may provoke severe AD for sustained periods of time. Based on our results, we suggest that blood pressure monitoring be incorporated during UDS in individuals with SCI, particularly in those with high-level autonomically-complete lesions. Blood pressure increases can be profound and highly symptomatic, and procedures should be in place to mitigate AD during UDS where appropriate. Of note, we showed a positive correlation between detrusor pressure and AD burden, supporting the notion (Furusawa et al., 2011) that there may be a dose-dependent relationship between stimulus and severity of AD. It is also important to note that the higher incidence of AD and larger increases in SAP compared to previous reports of AD during UDS (Huang et al., 2011; Koyuncu and Ersoz, 2017) are likely due to our use of continuous beat-to-beat recording, enabling all cardiovascular responses to be faithfully captured. The true magnitude of AD may be underestimated with intermittent cardiovascular monitoring because it may fail to capture sporadic increases in blood pressure. While we examined responses to UDS in the present study, it is likely that other medical and self-care procedures, particularly those associated with visceral stimuli, will also provoke similarly severe responses in susceptible individuals (Claydon et al., 2006; Lucci et al., 2020) and caregiver, healthcare practitioners, and individuals living with SCI should monitor for and treat episodes of AD in these situations.

We showed that the cardiovascular and cerebrovascular responses during UDS were more pronounced in those with autonomically-complete lesions, based on quantitative measurements of remaining sympathetic function inferred from markers of sympathetic vascular tone (LF SAP) and circulating levels of NE. These measures have also been shown previously to predict the severity of cardiovascular dysfunction such as the severity of AD, prevalence of OH, self-reported symptoms of OH and AD, risk for both atrial and ventricular arrhythmia, and the presence of abnormal heart rhythms (Claydon and Krassioukov, 2007; Ravensbergen et al., 2012; Sahota et al., 2012; Lucci et al., 2020; Lucci et al., 2021a; Lucci et al., 2021b), highlighting the utility of this approach in research, clinical and basic science settings. While both LF SAP and plasma NE were useful markers of autonomic function, LF SAP is non-invasive and simple to perform and thus may be more convenient for routine autonomic function assessment (Claydon and Krassioukov, 2007; Lucci et al., 2021a). Interestingly, despite its comprehensive *qualitative* approach, the ISAFSCI score did not predict or discriminate for either presence or severity of AD or symptoms of autonomic dysfunction, and this limitation of the ISAFSCI score has also been noted previously (Vaccaro et al., 2022). While we recognise the comprehensive approach of the ISAFSCI and its potential utility when screening for cardiovascular abnormalities after SCI, it does not discriminate between individuals with different severities of injury to cardiovascular autonomic pathways, highlighting the importance of additional quantitative measures of cardiovascular autonomic function after SCI.

We defined AD according to the current PVA (Consortium for Spinal Cord Medicine, 2002) and ISAFSCI criteria (Wecht et al., 2021) as a rise in SAP >20 mmHg from baseline. All but two participants met criteria for AD based on this definition, despite many of them having autonomically-incomplete lesions based on quantitative measures of autonomic function, and several individuals having low level lesions that are not compatible with the known physiology of AD. This raises the question as to whether the current definition of AD has appropriate sensitivity and specificity to faithfully discriminate pathophysiological episodes of AD from other physiological causes of increased blood pressure. Others have also noted challenges with the current definition of AD, proposing various additions that might increase the diagnostic value, including incorporation of DAP in AD classification (Kirshblum et al., 2021), the use of a larger minimum threshold for SAP increase in the definition of AD (Shergill et al., 2004), or requiring an increase in SAP of at least 20% from baseline (Karlsson, 1999). However, none of these methods were able to discriminate severity of AD based on autonomic injury in our participants, suggesting that some other component of AD quantification is needed to enhance discrimination.

During UDS, those with autonomically-complete injuries experienced decreases in HR associated with the hypertension triggered by UDS, a finding that was not seen in those with autonomically-incomplete lesions. Bradycardia (defined as HR < 60 bpm) would be expected during AD as the cardiac efferent arm of the baroreflex attempts to reduce HR and ameliorate the increase in blood pressure. This is supported by recent work confirming that episodes of AD are associated with a HR decrease and increased incidence of bradycardia (Yee et al., 2022). This could be an important component to consider in the criteria for AD. Blood pressure increases associated with exercise or stress responses would not be expected to be associated with bradycardia, but rather would be associated with constant or increased heart rates (Solinsky and Linsenmeyer, 2019). Incorporation of a heart rate component to the AD definition could help discriminate against increases in SAP seen during urodynamics that may be attributed to the nature of the test or patient discomfort. Certainly, a recent report also identified modest increases in blood pressure even in those with low level lesions during UDS that were ascribed to anxiety and stress associated with the procedure (Solinsky and Linsenmeyer, 2019). If we had included concurrent bradycardia in addition to the blood pressure rise in the definition of AD, 8/9 (89%) of those with autonomically-complete injuries but only 2/13 (15%) of those with autonomically-incomplete lesions would have met criteria for AD during UDS, increasing the accuracy of the current definition. This raises the question as to whether HR responses should be incorporated in the definition of AD.

Past work has revealed that autonomic injury is also associated with a higher incidence of OH, due to the inability to compensate for gravitational fluid shifts during position changes (Mathias, 2006). In individuals with high level autonomically-complete SCI, dynamic cerebral autoregulatory responses during orthostasis are diminished, and this was associated with more severe symptoms of OH (Sahota et al., 2012). We showed that AD symptom severity was positively correlated with the autonomic completeness of injury, and with the OH symptom severity and this is consistent with the pathophysiology of autonomic dysfunction after SCI, where individuals with autonomic injury experience both profound hypo- and hypertension regularly. These large fluctuations in blood pressure represent a significant cardiovascular health concern and are linked to earlier onset of cardiovascular disease (Currie et al., 2019; Sachdeva, Nightingale and Krassioukov, 2019), underscoring the need for comprehensive management of both the high and low blood pressure episodes that occur after high-level SCI.

The sustained and high blood pressures observed during UDS in those with autonomically-complete SCI were associated with an increased risk for both atrial (PWD variability) and ventricular arrhythmia (T_peak_-T_end_ variability). These results are consistent with past reports (Forrest, 1991; Claydon et al., 2006; Ravensbergen et al., 2012; Lucci et al., 2020), and likely represent a combination of cardiac remodelling secondary to loss of cardiac sympathetic drive after SCI, and the presence of autonomic conflict during AD (Collins, Rodenbaugh and DiCarlo, 2006; Lucci et al., 2021b). These data highlight consistent increases in these markers for arrhythmia risk seen in both acute and chronic SCI compared to reference data in controls (Ravensbergen et al., 2012; Lucci et al., 2021a) and recapitulate the observation that these markers are more severely impaired with autonomically-complete lesions (Ravensbergen et al., 2012) and increase further with paroxysmal sympathetic activation (Lucci et al., 2021b). Interestingly, QTVI data were closer to zero in all participants than previous reports in healthy controls (Ravensbergen et al., 2012), confirming greater susceptibility to ventricular arrhythmia. We expected QTVI to be more positive during episodes of AD triggered by UDS, but this did not achieve statistical significance, and this may reflect that these analyses are less robust in recordings with fewer samples. While we were not able to evaluate the incidence of cardiac arrhythmia during AD in the present study, arrhythmia and palpitations during AD are well documented in the literature (Forrest, 1991; Claydon et al., 2006; Collins, Rodenbaugh and DiCarlo, 2006; Lucci et al., 2020).

As noted above, we and others have previously shown that cerebrovascular control is diminished during orthostatic stress and mild thermal stress in individuals with high level autonomically-complete SCI (Sahota et al., 2012; van der Scheer et al., 2018). Recent work suggests that cerebrovascular responses are not equally attenuated among increases and decreases in pressures, with cerebral autoregulatory responses being better adapted to increases in systemic pressures than to hypotensive episodes (Numan et al., 2014; Brassard et al., 2017). This discrepancy in autoregulatory buffering could explain the robust cerebrovascular resistance responses and intact static and dynamic autoregulation observed during the profound increases in blood pressure seen during UDS, even in those with severe AD and high-level autonomically-complete lesions (Claassen et al., 2016). Indeed, while buffering was not perfect, MCAv did not significantly increase during UDS, and, despite low coherence, parameters of gain derived from both static and dynamic cerebral autoregulatory measures were similar to previous normative data in healthy controls (Claassen et al., 2016), even during extreme hypertension. Recent work in cerebrovascular research has suggested a change to the dogmatic thinking of cerebral autoregulation (Brassard et al., 2021; Claassen et al., 2021) and it is possible that these data further highlight that autoregulatory processes are not equal across the autoregulatory range. It is interesting that the individuals with autonomically-complete lesions in this study were able to buffer cerebral blood flow remarkably well, even in the face of very high blood pressures associated with AD, and it is not clear whether these pressures would typically be as well tolerated in those not exposed to them on a regular basis, i.e., whether preconditioning through repeated exposure to episodes of AD during activities of daily living plays a role. It is likely, however, that this ability to buffer cerebral blood flow during extreme hypertension would be protective, and might explain why, although AD is associated with adverse cerebrovascular events (Pan et al., 2005; Sachdeva, Nightingale and Krassioukov, 2019), they are not in fact more common during episodes of AD. These findings should, however, be interpreted with caution because of the known heterogeneity of SCI, small sample size, and low coherence observed in the dynamic autoregulation measures.

This study was carefully conducted, with beat-to-beat cardiovascular and cerebrovascular monitoring, extensive quantitative and qualitative assessment of remaining autonomic function, as well as symptom evaluation, during a controlled stimulus for AD. The primary limitation of the study is that it is likely that UDS testing (which requires participants to be partially unclothed and is somewhat unpleasant) provokes a sympathetically-mediated stress response, at least in some participants, much akin to “white-coat hypertension”. This may explain the modest increase in blood pressure during UDS in those with autonomically-incomplete lesions, particularly because, unlike with AD, the blood pressure elevation was not associated with bradycardia. It is also possible that this form of stress-induced or white-coat hypertension may be larger in individuals with SCI than in the able-bodied, because adrenoreceptor hypersensitivity below the lesion level may contribute to exaggerated vasoconstrictor responses and large blood pressure increments (Solinsky and Linsenmeyer, 2019). It is also possible that this stress response contributes to the blood pressure rise in those with high-level autonomically-complete lesions, although based on the associated bradycardia it is likely that the AD mechanism is the dominant cause of the hypertensive response elicited in these individuals.

## 5 Conclusion

UDS is an unavoidable aspect of care post-SCI. We have shown that autonomic dysfunction is severe during UDS, especially in individuals with autonomically-complete SCI. In addition to profound changes to blood pressure and HR, autonomically-complete injury is associated with increased risk for cardiac arrythmia and greater symptom burden during the procedure. However, the cerebral circulation was relatively well protected against the severe hypertension of AD, and this may serve to be neuroprotective. Appropriate quantification of cardiovascular autonomic dysfunction after SCI is a priority, and this work further highlights the utility of LF SAP as a discrete, quantitative marker of autonomic completeness of injury. While the ISAFSCI may be a useful clinical tool to screen for cardiovascular autonomic abnormalities, it was not able to discriminate autonomic severity of injury or risk for AD. This study also highlights the benefits of incorporating HR responses in the definition of AD, particularly in scenarios where stress or exercise stimuli might provoke blood pressure increases that could be mistaken for AD. Together, these data will inform the diagnosis and management of autonomic dysfunction for individuals living with SCI, aiding risk stratification, reducing symptom burden, decreasing cardiovascular morbidity and mortality, and improving quality of life.

## Data Availability

Due to legal and ethical restrictions, data cannot be made publicly available. Data will be made available upon request; however, only aggregated data may be in the public domain according to the stipulations from our research ethics board with respect to the maintenance of confidentiality. Additional published or public analyses would only be permitted with ethics approval for secondary data access, and only with aggregated analyses.
