# Supplementary material for: Cardiovascular and cerebrovascular responses to urodynamics testing after spinal cord injury: The influence of autonomic injury
Source: Front Physiol. 2022 Sep 16;13:977772. doi: 10.3389/fphys.2022.977772 (PMC9525190; doi:10.3389/fphys.2022.977772)
Supplement: Supplementary file 1 [file Table1.DOCX]

**Supplemental Table 1. Cardiovascular and cerebrovascular responses to urodynamics testing over time.**

|  | **Baseline** | **Max AD during UDS** | **Recovery** | **Time** | **Completeness** | **Interaction** |
| --- | --- | --- | --- | --- | --- | --- |
| **HR (bpm)**  Autonomically-complete  Autonomically-incomplete | 62.46±2.86  69.88±2.72 | 56.52±4.63^R^*  67.33±3.03 | 60.87±1.68  66.76±2.07 | **0.043** | 0.075 | 0.110 |
| **SAP (mmHg)**  Autonomically-complete  Autonomically-incomplete | 117.57±4.96  125.13±3.54 | 207.07±12.31^BR^*  165.93±5.34^BR^ | 122.66±6.30  133.15±5.00 | **<0.001** | 0.232 | **<0.001** |
| **DAP (mmHg)**  Autonomically-complete  Autonomically-incomplete | 66.83±1.48  68.47±2.49 | 99.15±4.35 ^BR^*  85.26±3.36 ^BR^ | 70.69±2.97 71.38±3.56 | **<0.001** | 0.322 | **0.007** |
| **SV (mL)**  Autonomically-complete  Autonomically-incomplete | 101.15±10.59  89.49±6.01 | 102.01±13.03  102.79±11.03 | 104.25±6.70  94.81±6.57 | 0.488 | 0.671 | 0.599 |
| **CO (L/min)**  Autonomically-complete  Autonomically-incomplete | 6.35±0.68  6.40±0.31 | 5.18±0.73  6.70±0.58 | 5.64±0.80  6.26±0.39 | 0.375 | 0.225 | 0.189 |
| **TPR (mmHg·min·L^-1^)**  Autonomically-complete  Autonomically-incomplete | 15.22±2.77  13.96±0.58 | 23.17±3.13^BR^*  16.80±0.98 | 12.01±2.15  15.42±1.20 | **<0.001** | 0.437 | **0.021** |
| **CBFV_M_ (cm·s^-1^)**  Autonomically-complete  Autonomically-incomplete | 63.63±3.01  65.30±2.19 | 75.43±7.19  71.55±3.53 | 68.81±5.64  68.29±3.53 | 0.089 | 0.788 | 0.786 |
| **CBFV_S_ (cm·s^-1^)**  Autonomically-complete  Autonomically-incomplete | 102.65±4.40  104.41±2.91 | 115.17±3.15  110.13±3.52 | 110.07±9.15  105.88±4.75 | 0.146 | 0.628 | 0.755 |
| **CBFV_D_ (cm·s^-1^)**  Autonomically-complete  Autonomically-incomplete | 41.14±3.32  41.87±1.85 | 51.07±6.18  46.89±3.55 | 45.16±3.57  44.59±2.62 | 0.108 | 0.642 | 0.770 |

Data are presented as mean±SEM. Statistical significance: * represents statistical difference between groups; ^B^ represents statistical difference from baseline condition; ^R^ represents statistical difference recovery condition. Statistical significance (p-values) are also presented for the main effects of time point, subgroup classification based on autonomic completeness of injury, and their interactions where bold text indicates significant main effect and italicised text indicates analysis that did not quite meet criteria for statistical significance. Abbreviations: AD, autonomic dysreflexia; UDS, urodynamics test; HR, heart rate; SAP, systolic arterial pressure; DAP, diastolic arterial pressure; SV, stroke volume; CO, cardiac output; TPR, total peripheral resistance; CBFV_M_, mean cerebral blood flow velocity CBFV_S_, systolic cerebral blood flow velocity; CBFV_D_, diastolic cerebral blood flow velocity.
